# Protein Data Bank (PDB) Archive: a new architecture (beta) for scalable, PDBx/mmCIF-based data distribution

**DOI:** 10.1107/S2059798326006194

**Published:** 2026-07-17

**Authors:** Zukang Feng, Balakumaran Balasubramaniyan, Gert-Jan Bekker, Jose M. Duarte, Vladimir Guranovic, Jeremy Henry, Sreenath S. Nair, Ezra Peisach, Dennis W. Piehl, Aditya Pingale, James Smith, Brinda Vallat, Reiko Yamashita, Arthur Zalevsky, Kyle Morris, Jeff Hoch, Genji Kurisu, Sameer Velankar, Stephen K. Burley, Jasmine Y. Young

**Affiliations:** a RCSB Protein Data Bank Rutgers, The State University of New Jersey Piscataway New Jersey USA; b PDBe EMBL–European Bioinformatics Institute HinxtonCB10 1SD United Kingdom; c Protein Data Bank Japan, Institute for Protein Research The University of Osaka Osaka565-0871 Japan; d RCSB Protein Data Bank, San Diego Supercomputer Center University of California San Diego San Diego California USA; e RCSB Protein Data Bank University of California San Francisco San Francisco California USA; f EMDB EMBL–European Bioinformatics Institute HinxtonCB10 1SD United Kingdom; g BMRB UConn Health Farmington Connecticut USA; h Protein Data Bank Japan Protein Research Foundation Minoh Osaka562-8686 Japan; i Institute for Quantitative Biomedicine Rutgers, The State University of New Jersey Piscataway New Jersey USA; j Rutgers Cancer Institute Rutgers, The State University of New Jersey Piscataway New Jersey USA; k Rutgers Artificial Intelligence and Data Science Collaboratory Rutgers, The State University of New Jersey Piscataway New Jersey USA; l Department of Chemistry and Chemical Biology Rutgers, The State University of New Jersey Piscataway New Jersey USA; University of Florida, USA

**Keywords:** Protein Data Bank, PDB, macromolecular structure, mmCIF, structural biology, data archive

## Abstract

With the continuous growth of the Protein Data Bank archive, the Worldwide Protein Data Bank (wwPDB) partnership anticipates that the entire complement of possible four-character PDB accession codes (for example 1ABC) will be exhausted by 2028. wwPDB is, therefore, revising the PDB accession code (PDB ID) to 12 characters by extending its length and prepending ‘pdb_’ (for example pdb_1000axyz) in lower case. This change will enable the robust detection of references to PDB entries in published literature. On or about July 21st 2027, the PDB will convert to releasing entries with extended PDB IDs only, which will not be compatible with the legacy PDB format. A beta version of the PDB Archive (PDB Beta Archive) is now available to help communities adapt to and embrace the extended PDB IDs and PDBx/mmCIF format during a transition phase. All files in the current PDB archive are reorganized in the Beta Archive with extended PDB IDs (including file naming and directories) on an entry-level basis, mirroring the data organization of the PDB Versioned Archive. wwPDB encourages scientific journals, PDB community members and users to transition to the PDBx/mmCIF format and adopt the new PDB ID format as early as possible. The PDB Beta Archive will replace the current public archive, and 12-character PDB IDs will be solely assigned to all newly deposited PDB entries.

## Abstract 2

wwPDB has extended PDB IDs to 12 characters with a prefix ‘pdb_’ followed by eight alphanumeric characters in lower case (for example pdb_1000axyz). A beta version of the PDB Archive (https://files-beta.wwpdb.org), with extended PDB IDs and an improved directory structure, is now available to help communities adopt extended PDB IDs and the PDBx/mmCIF format.

## 1. Introduction

The Protein Data Bank (PDB) was established in 1971 (Protein Data Bank, 1971 ▸; wwPDB Consortium, 2019 ▸) as the first open-access digital data resource in biology, initially comprising just seven X-ray crystal structures of proteins. Today, it houses more than 250 000 experimentally determined three-dimensional (3D) structures of biological macromolecules that are freely used by many millions of PDB data consumers worldwide (Berman, 2008 ▸; Berman & Burley, 2025 ▸). This wealth of information serves as a cornerstone for basic and applied research and education endeavours across fundamental biology, biomedicine, biotechnology, bioengineering and energy sciences.

The Worldwide Protein Data Bank partnership (wwPDB, https://wwpdb.org; Berman *et al.*, 2003 ▸) currently consists of five full members (RCSB Protein Data Bank or RCSB PDB, Protein Data Bank in Europe or PDBe, Protein Data Bank Japan or PDBj, Biological Magnetic Resonance Data Bank or BMRB, and Electron Microscopy Data Bank or EMDB) and one associate member (Protein Data Bank China or PDBc; Xu *et al.*, 2023 ▸). The wwPDB partners jointly manage three Core Archives (wwPDB Consortium, 2019 ▸), including PDB (archiving atomic coordinates, crystallographic data and NMR chemical shifts/restraints), EMDB (archiving 3DEM density maps) and BMRB (archiving NMR experimental data), all the while adhering to the FAIR (Findability, Accessibility, Interoperability, Reusability; Wilkinson *et al.*, 2016 ▸) and FACT (Fairness, Accuracy, Confidentiality, Transparency; van der Aalst *et al.*, 2017 ▸) principles emblematic of responsible data stewardship.

Stemming from advances in technology and methodologies, the number, complexity and size of biomolecular structural studies have grown rapidly, posing challenges for data archiving. Advances across structure-determination pipelines include improvements in sample preparation and delivery, the development and refinement of data-collection instruments and detectors, innovations in data processing and analysis algorithms, and increased computational power. Rapidly evolving experimental approaches, such as cryo-electron microscopy (cryo-EM), which has improved in average resolution from >14 Å to a best resolution of ∼1 Å since 2013 (Kühlbrandt, 2014 ▸), X-ray free-electron lasers and serial crystallography for time-resolved studies, integrative/hybrid methods (IHM) that combine multiple experimental and computational techniques (Vallat *et al.*, 2025 ▸), and high-throughput screening of protein libraries with small molecules have further accelerated progress. Advances in experimental pipelines, coupled with computational and AI-driven modelling, and the structural biology community’s commitment to open data sharing, have driven substantial growth of the PDB archive.

The legacy PDB file format (Bernstein *et al.*, 1977 ▸), based on the 80-column Hollerith punch-card standard, has limitations on the number of columns and characters, including identifiers for molecules described in the Chemical Component Dictionary (CCD IDs; Westbrook *et al.*, 2015 ▸) and PDB accession codes (PDB IDs). Previously, wwPDB took steps to address the limitation in the PDB legacy format. Initially, historically large ribosome structures were split into several PDB files due to limits on the number of atoms and/or polymer chain IDs supported by the legacy PDB format, which hinder the ability for users to gain structural insight into the full structure and wwPDB realization of FAIR principles. In 2014, all large structures that were previously split into multiple PDB entries were combined into single files formatted according to the PDB Exchange/macromolecular Crystallographic Information Framework (PDBx/mmCIF; Fitzgerald *et al.*, 2005 ▸; Westbrook *et al.*, 2022 ▸). Another limitation of the legacy PDB format is the length restriction of residue names to three characters, as defined in the CCD. Recently, all three-character CCD IDs were consumed. Newly deposited novel ligands are now assigned five-character CCD IDs, which are no longer compatible with the legacy PDB file format. Finally, with the continued growth of PDB depositions, wwPDB anticipates that four-character PDB IDs will be fully consumed by 2028. wwPDB has given careful consideration in defining the new extended PDB ID code to both facilitate text mining and support deposition growth.

For more than a decade, wwPDB has enhanced the content of the PDB archive by introducing innovative, new features: PDB Archive (https://files.wwpdb.org) provides the historical archive, PDB Versioned Archive (https://files-versioned.wwpdb.org) enables the distribution of different versions of PDB entries and PDB NextGen Archive (https://files-nextgen.wwpdb.org) distributes enhanced information available in the individual PDB entries, to support the diverse needs of our very broad user community. PDB Archive is the primary repository, which distributes the latest versions of all data files for each PDB entry.

Versioning of PDB entries (stored in the PDB Versioned Archive) was introduced in 2017 and distributes the latest versions of every major version of structure model files for each PDB entry. Necessary modifications made to a PDB entry after its initial release can be either ‘major’ or ‘minor’. Updates to atomic coordinates, polymer sequence or chemical description in the coordinate file trigger a major version increment. Other changes to the metadata in the coordinate file are identified with a minor version increment. In both cases, the originally issued PDB accession code is retained. To enable tracking of changes between versions, new revision data categories were defined in the PDBx/mmCIF dictionary (https://mmcif.wwpdb.org/dictionaries/mmcif_pdbx_v50.dic/Groups/audit_group.html). The revision trail is provided within the PDBx/mmCIF-formatted coordinate files. This PDB Versioned Archive has adopted the new extended PDB ID in the directory and file naming in the archive layout.

The PDB NextGen Archive (Choudhary *et al.*, 2024 ▸) was created in 2023 as a prototype to provide enriched annotations for the latest version of each entry compared with the PDB Archive. This PDB NextGen Archive incorporates additional metadata annotations from external data resources within the structure model files in the PDB Archive. For example, up-to-date sequence annotations from the SIFTS resource (Dana *et al.*, 2019 ▸) to external resources such as UniProt, SCOP2 and Pfam are provided in this newer archive. The layout of the NextGen Archive follows the same layout as the Versioned Archive, using the extended PDB ID in the directory and file naming. To render the PDB Archive more sustainable while enhancing accessibility and scalability, wwPDB has restructured a beta version of the PDB Archive with data reorganization based on the new extended PDB ID at an entry-level basis.

Herein, we describe the beta version of the PDB Archive (henceforth referred to as the PDB Beta Archive), incorporating the new updates, with the goal of supporting PDB users as they transition to the extended PDB ID and PDBx/mmCIF format. Eventually, the PDB Beta Archive will replace the current PDB Archive once 12-character PDB IDs are issued exclusively. Users are strongly encouraged to adapt to these changes in PDB data layout and content as soon as possible.

## 2. Methods

### 2.1. Extended PDB ID format

To support the ongoing growth of the PDB archive and make it more FAIR, wwPDB has extended the PDB accession code to be 12 characters in length with the prefix ‘pdb_’ (*e.g.* pdb_1000axyz) in lower case. The prefix ‘pdb_’ in the extended PDB IDs will facilitate text mining of PDB entries referenced in the published literature. PDB entries bearing only extended PDB IDs (*i.e.* those that do not have a corresponding four-character ID) are incompatible with legacy PDB format and will be distributed only in PDBx/mmCIF and PDBML (XML) formats.

Since the late 1990s, the PDB Archive has provided entries in PDBx/mmCIF format (Westbrook *et al.*, 2022 ▸; https://mmcif.wwpdb.org). The PDBx/mmCIF framework was built atop the self-validating Data Definition Language Version 2 (DDL2) dictionary (Westbrook *et al.*, 2005 ▸), which defines metadata (*e.g.* data types, allowed ranges, controlled vocabularies) and parent–child relationships. The PDBx/mmCIF dictionary explicitly defines all relationships between data items (*e.g.* atom and residue identifiers), which enables software applications to evaluate and validate referential integrity for any biostructure data represented in this form (*e.g.* within a PDB entry) and maps information between two related categories (*e.g.* the sample sequences and the observed sequences from the model coordinates). The PDBx/mmCIF dictionary is fully extensible and both human- and machine-readable, allows the accommodation of metadata from new and increasingly complex experimental methods such as serial crystallography, X-ray free-electron lasers and integrative/hybrid methods (Sali, 2021 ▸), and helps to ensure that new metadata items conform to data standards.

Since 2014, PDBx/mmCIF has become the master PDB archival format (Berman *et al.*, 2014 ▸) when the OneDep system (Young *et al.*, 2017 ▸) was launched for deposition, validation (Feng *et al.*, 2021 ▸; Gore *et al.*, 2017 ▸) and biocuration (Young *et al.*, 2018 ▸) of incoming PDB and EMDB entries. The PDBx/mmCIF format has no limitations. It can accommodate ribosomes and other large structures in a single file and supports better realization of FAIR principles. This format is extendable to support new methods development and the increasing complexity of structures. PDBx/mmCIF can be programmatically parsed, searched and edited, and is managed by the wwPDB and the mmCIF Working Group consisting of community experts (https://www.wwpdb.org/task/mmcif; Westbrook *et al.*, 2022 ▸).

All PDB entries, including IHM structures, issued with four-character PDB IDs have been assigned an extended PDB ID by prepending ‘pdb_0000’ to the four-character ID (*e.g.* PDB ID 1ABC will have the extended PDB ID pdb_00001abc). For such entries, the PDB Digital Object Identifier (DOI) format with ‘pdb’ prefix, 10.2210/pdb<four-character PDB ID>/pdb, remains unchanged, as necessary to respect FAIR data practices (*e.g.* 10.2210/pdb1abc/pdb). Both the four-character and extended PDB ID are stored in the database_2 category of the PDBx/mmCIF model file:
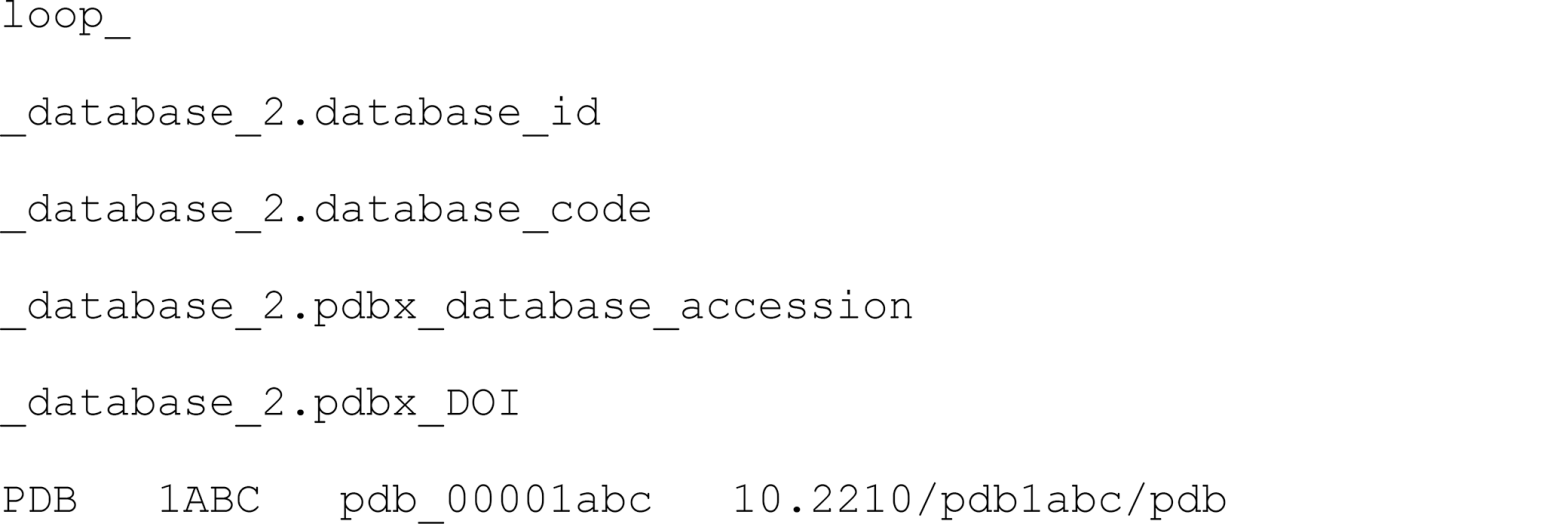


PDB entries identified solely with extended PDB IDs issued (*e.g.* pdb_1000axyz) will have the same 12-character PDB ID in both the _database_2.database_code and _database_2.pdbx_database_accession data items. Such entries will have a new PDB DOI format based on the extended PDB ID: 10.2210/<12-character PDB ID>/pdb (*e.g.* 10.2210/pdb_1000axyz/pdb):
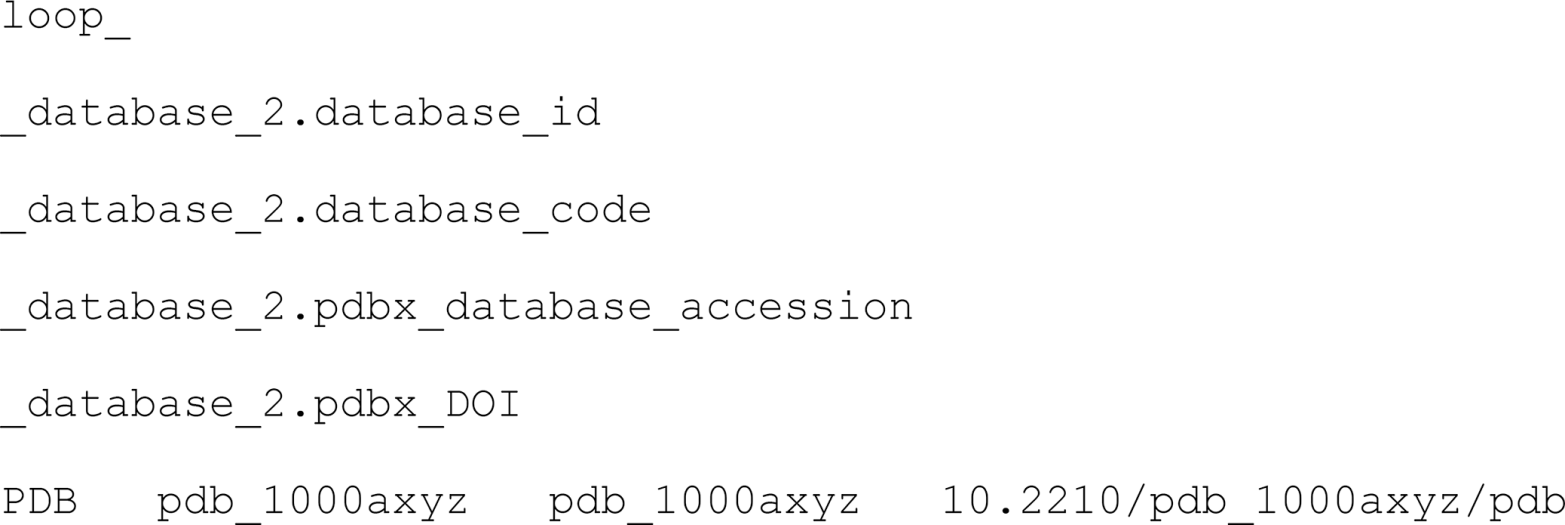


### 2.2. Creation of PDB Beta Archive with extended PDB IDs

A new PDB Beta Archive was created to allow better presentation of all the abovementioned changes and enriched annotations, and help users adapt to extended PDB IDs and the PDBx/mmCIF format. This PDB Beta Archive contains PDB entries in PDBx/mmCIF and PDBML formats, with extended PDB IDs in the datablock name. It is organized on an entry-level basis (*e.g.* all associated experimental and validation files for a particular entry are contained within the same directory) with extended PDB IDs used in the file and directory naming.

Entry data files (*e.g.* structure model and structure-factor files) with extended PDB IDs in PDBx/mmCIF-formatted files and file naming are generated by the OneDep release module alongside the data files with four-character PDB IDs in PDBx/mmCIF-formatted files and file naming, which will remain accessible in the current main archive when four-letter PDB ID code issuance is discontinued in July 2027. The wwPDB weekly update process was modified to create the PDB Beta Archive alongside the current PDB Archive. Both PDB Beta and the current main archives are updated in parallel each week and made available every Wednesday at 00:00 UTC.

The PDB-IHM data pipeline and workflows were extended to generate updated files for the Beta Archive during the weekly update process, alongside the PDB-IHM branch of the current main archive. The model files and validation reports are organized at the entry level and follow the 12-character extended PDB ID naming convention, as for the PDB Beta Archive.

## 3. Results

A new PDB Beta Archive is available at https://files-beta.wwpdb.org to help communities adapt to and adopt extended PDB ID and PDBx/mmCIF format during the transition phase. A documentation page, https://www.wwpdb.org/ftp/pdb-beta-ftp-sites, with download instructions (including a script and URL short links) is provided to help users adapt to the new file organization and formats in the PDB Beta Archive. Existing legacy PDB-format files are retained and maintained wherever possible in this archive. Once four-character PDB IDs are consumed, newly deposited PDB entries, including the IHM structures, will only be issued extended PDB IDs and will only be distributed in PDBx/mmCIF and PDBML formats.

### 3.1. Data structure and file-naming conventions

**Figure 1:**
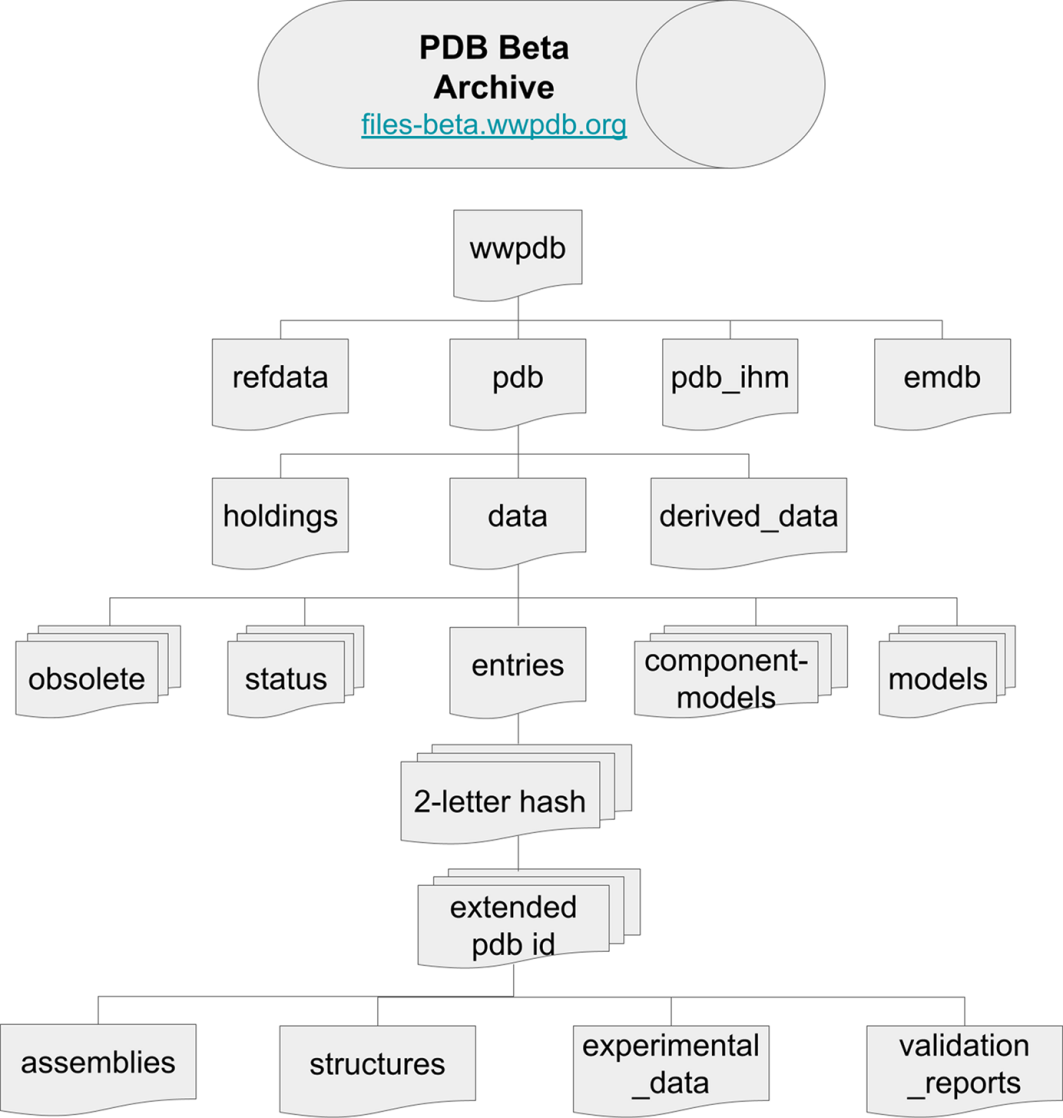
Organization of data within the PDB Beta Archive. A /wwpdb directory is introduced under the /pub top directory. Small-molecule data are stored in the /refdata/ subdirectory. PDB data, derived data and PDB holding files, which are inventory data files that offer a quick overview of data in the archive, are stored under the /pdb/ subfolder. 3D structure atomic coordinate model files, experimental data files, assembly files and validation reports associated with each entry are stored under its extended PDB ID subfolder under a hash directory. The /pdb_ihm/ folder consists of /data/ and /holdings/ subfolders corresponding to IHM structures (not shown) (Johnson *et al.*, 2024 ▸; Baldwin *et al.*, 2024 ▸).

A high-level subdirectory (/wwpdb/) under the /pubtop directory has been introduced within the PDB Beta Archive (https://files-beta.wwpdb.org/pub/wwpdb/). Within this /wwpdb subdirectory are subdirectories for each data type, including small-molecule or CCD data (/refdata/), PDB data (/pdb/), IHM data (/pdb_ihm/) and EMDB data (/emdb/). Fig. 1 ▸ illustrates the tree structure data organization of the PDB Beta Archive. Within the /pub/wwpdb/pdb subdirectory are PDB data, derived data (*i.e.* wwPDB-computed data files based on PDB data *etc.*) and PDB holding files (*i.e.* inventory index files that offer a quick overview of archival contents).

All data files associated with a given entry, such as structure model, experimental data, assemblies and validation reports, are stored on an entry-level basis using the extended PDB ID as the folder name under a hash directory, https://files-beta.wwpdb.org/pub/wwpdb/pdb/data/entries/. The two-character hash is generated from the penultimate two characters of the PDB code (*i.e.* the second and third characters from the last character, https://files-beta.wwpdb.org/pub/wwpdb/pdb/data/entries/<two-letter-hash>/<pdb_accession_code>/<entry_data_file_names>). For example, PDB entry pdb_1abc5**67**8 will be stored under the /**67**/ subdirectory. This measure will maintain consistency with the current PDB archive: PDB entry 1ABC (with extended PDB ID pdb_00001abc) is stored under /ab/.

File naming has been standardized, such that the file type is used for the extension. For example, the file naming for structure-factor data was changed from r116dsf.ent.gz to pdb_0000116d-sf.cif.gz with a .cif extension as the file type and from pdb318d.ent.gz to pdb_0000318d.pdb.gz with a .pdb extension as the file type for the legacy PDB-formatted coordinate file.

### 3.2. File access and short links

The PDB Beta Archive is updated every Wednesday at 00:00 UTC. wwPDB has provided various download protocols, including https and rsync:

wwPDB: https://files-beta.wwpdb.org, rsync://rsync-beta.wwpdb.org.
RCSB PDB (US): https://files-beta.rcsb.org, rsync://rsync-beta.rcsb.org.
PDBe (UK): https://ftp.ebi.ac.uk/pub/databases/wwpdb/.
PDBj (Japan): ftp://ftp-beta.pdbj.org, https://files-beta.pdbj.org, rsync://rsync-beta.pdbj.org.

To help PDB users readily access data files in the PDB Beta Archive, wwPDB has provided download short links that obviate the need for prior knowledge of file paths. Table 1 ▸ lists the file paths for each data type and provides download short links. To use these short links, simply replace the example ID, pdb_00001abc, with a real PDB ID.

To perform a batch download of all PDB entries of a file type or format from the PDB Beta Archive, wwPDB also provides a download script, https://cdn.rcsb.org/wwpdb/docs/BetaArchiveBatchDownloader.py. It uses an asynchronous aiohttp library to download multiple files in parallel. It requires Python 3.8 or higher, plus the *aiofiles* and *aiohttp* packages. Detailed instructions can be found at https://www.wwpdb.org/ftp/pdb-beta-ftp-sites#downloadPor.

## 4. Discussion

### 4.1. Communication/community engagement

The development and maintenance of PDBx/mmCIF in collaboration with the macromolecular crystallography (MX) community led to the formation of the wwPDB mmCIF Working Group. The Working Group was initially established in 2011 to enable the use of PDBx/mmCIF-format files in major macromolecular crystallographic software tools, and to provide recommendations on format extensions required for deposition of larger structures to the PDB. This working group meets regularly with wwPDB leadership and select subject-matter experts to guide dictionary content and promotes the standard among structural biology methods developers.

PDBx/mmCIF has been adopted by the community broadly including major refinement-software packages [*Phenix* (Adams *et al.*, 2010 ▸), *REFMAC* (Murshudov *et al.*, 2011 ▸), *Global Phasing* (Blanc *et al.*, 2004 ▸; Bricogne *et al.*, 2009 ▸)], visualization programs [*PyMOL* (DeLano, 2002 ▸), *ChimeraX* (Meng *et al.*, 2023 ▸), *CCP*4*mg* (McNicholas *et al.*, 2011 ▸), Mol* (Sehnal *et al.*, 2021 ▸) *etc.*], *pdb_extract* tool (Yang *et al.*, 2004 ▸), *PDBj CIF Editor* and various mmCIF parsers (*e.g.py-mmcif*, *Gemmi*, *iotxb.cif*, *mmcif_pdbx*). The adoption of PDBx/mmCIF format was extended to other communities with the development of *ModelCIF* (Vallat *et al.*, 2023 ▸) for computed structure models and *IHMCIF* (Vallat *et al.*, 2024 ▸) for integrative/hybrid structures. In 2019, PDBx/mmCIF was made the mandatory format for the deposition of macromolecular crystallography structures to the PDB (Adams *et al.*, 2019 ▸).

wwPDB continues to engage with the editors of the top 25 scientific journals that publish most macromolecular structures deposited to the PDB to inform them of the extended PDB ID format (in text, tables and data-availability statements) and corresponding PDB DOI links in their pipelines. Early adoption will contribute to the long-term sustainability and interoperability of 3D biostructure data across the scientific community.

For all extant PDB entries with four-character PDB IDs, PDB DOIs are formatted as 10.2210/pdb[four-character_PDB_ID]/pdb and can be used to link to the corresponding wwPDB DOI landing page. For example, the PDB entry with ID 8Y9M (and extended PDB ID pdb_00008y9m) has the DOI https://doi.org/10.2210/pdb8y9m/pdb. Importantly, previously issued DOIs are persistent and will remain unchanged, following FAIR data best practices.

Once the issuance of four-character PDB IDs ceases in July 2027, all new PDB entries, including IHM structures in PDB-IHM, will only be issued extended PDB IDs, and their corresponding PDB DOIs will be formatted as 10.2210/[extended_PDB_ID]/pdb, leading to the corresponding wwPDB DOI landing page. For example, a PDB entry with ID pdb_10001xyz will have the DOI https://doi.org/10.2210/pdb_10001xyz/pdb.

wwPDB has also reached out broadly to technically diverse research and technology communities, including structural biology, visualization tools, bioinformatics, cheminformatics, computational structure prediction and third-party database resources that utilize PDB data. We are strongly encouraging them to transition to extended PDB ID and PDBx/mmCIF formats through individual email communication, news announcements via various community mailing lists, poster and oral presentations at professional society meetings, and offering webinars and virtual office hours focused on these topics.

### 4.2. Training materials and tools for adoption

Many training materials have been created to help users learn about extended PDB IDs and PDBx/mmCIF, including an mmCIF user guide, FAQ pages, recorded webinars and tutorials. Resource tools for mmCIF parsers and file-format conversion are available at https://mmcif.wwpdb.org/docs/software-resources.html for users to extract information from PDBx/mmCIF files or convert PDBx/mmCIF to PDB format, if needed, until all users have adopted PDBx/mmCIF. For example, the wwPDB software tool *Maxit* can be used to convert PDBx/mmCIF to PDB format for PDB entries with four-character PDB IDs and without five-character CCD IDs. Alternatively, a community software tool, such as *Gemmi*, can convert PDBx/mmCIF to PDB format with minimal support for metadata and accession codes. For database users, a *DB-Loader* tool for loading data from PDBx/mmCIF files into a database is available for user adoption at https://sw-tools.rcsb.org/apps/DB-LOADER/index.html. *Mine*2*Updater* can be used to update a PostgresSQL database with weekly PDB releases: https://gitlab.com/pdbjapan/mine2updater/.

wwPDB also provides a CIF editor (see Table 2 ▸) that allows users to prepare and modify their own PDBx/mmCIF files. In addition, the *pdb_extract* tool allows PDB depositors to generate a PDBx/mmCIF file with common metadata that can be used to prepare a complete PDBx/mmCIF file for depositions via OneDep. wwPDB partners, and the software developers (CCP4, Phenix, CCP-EM, Global Phasing and others), are actively working to support the generation of PDBx/mmCIF files for data deposition. Table 2 ▸ provides information regarding available resources, and where users can access them to learn and utilize these tools to work with PDBx/mmCIF format files.

## 5. Conclusion and future perspectives

PDBx/mmCIF is the file format and archival data standard of the PDB. Its flexible and infinitely extensible format allows the accurate representation of large structures, while supporting new/evolving structure-determination methodologies. It is both human- and machine-readable. PDBx/mmCIF-format file contents can be parsed programmatically, or using three-dimensional visualization tools. As the PDB archive grows, PDBx/mmCIF- and PDBML-format files will become the exclusive means of distributing atomic coordinate information and related metadata.

Many resources have been made available to assist users in transitioning to extended PDB ID and PDBx/mmCIF. wwPDB is committed to providing additional resources, including webinars and virtual office hours, as needed for a smooth transition.

### 5.1. Timeline for user adoption

The PDB Beta Archive will become the PDB main core archive in July 2027. The https://files-beta.wwpdb.org URL will point to the main archive (https://files.wwpdb.org) for some time. Thereafter, the PDB Beta Archive URL (not the PDB Beta Archive) will be retired. PDB entries with extended PDB IDs only will not be compatible with the legacy PDB format. wwPDB strongly encourages scientific journals, PDB depositors and the PDB user community writ large to transition to the PDBx/mmCIF format and adopt extended PDB IDs as soon as possible.

When providing extended PDB IDs to scientific journals and citing extended PDB IDs in submitted manuscripts, all 12 characters, including the prefix ‘pdb_’, should be included (in lower case). After mid-2027, scientific journals should be routinely accepting 12-character extended PDB IDs with their new DOI format (*e.g.* PDB ID pdb_10001xyz will have DOI https://doi.org/10.2210/pdb_10001xyz/pdb), while retaining the existing DOI format for PDB entries with four-character PDB IDs or those starting with pdb_0000 (*e.g.* PDB ID 8Y9M or pdb_00008y9m will have the DOI https://doi.org/10.2210/pdb8y9m/pdb) for accurate cross-referencing in electronic manuscripts. (N.B. DOIs for existing PDB entries with legacy four-character PDB IDs have not been redefined, because DOIs are intended to be persistent as per the best FAIR data practices.)

### 5.2. Future plans for PDB archive management

Maintaining multiple PDB archives (PDB, Versioned and NextGen) represents a substantial challenge for the wwPDB. wwPDB addresses this challenge by creating a more sustainable PDB Beta Archive, which is structured to be scalable and extensible. This beta archive will replace the current PDB archive. Looking ahead, wwPDB envisions enhanced support for fragment-screening and time-resolved studies, which require grouping multiple PDB structures and additional metadata to describe overall characteristics of the experimental study. After the PDB Beta Archive becomes the PDB main core archive, this scalable archive will be augmented with content from the NextGen archive, thereby providing users with access to additional enriched annotations and the possibility of grouping multiple related entries. This PDB main core archive has been designed such that it can also be extended to house the versioned archival files, currently held in the Versioned archive. wwPDB partners welcome constructive feedback on potential improvements to the PDB Beta Archive for appropriate consideration.

## Figures and Tables

**Table 1:** PDB Beta Archive data organization and download short links

Data types	File formats	Location	Short link
Atomic coordinates	PDBx/mmCIF, PDBML and PDB	https://files-beta.wwpdb.org/pub/wwpdb/pdb/data/entries/[two-letter-hash]/[extended-PDB-ID]/structures/	https://files-beta.wwpdb.org/download/[extended PDB ID].[extension].gz, *e.g.* https://files-beta.wwpdb.org/download/pdb_00001abc.cif.gz
X-ray data: structure factors	PDBx/mmCIF	https://files-beta.wwpdb.org/pub/wwpdb/pdb/data/entries/[two-letter-hash]/[extended-PDB-ID]/experimental_data/	https://files-beta.wwpdb.org/download/[extended PDB ID]-sf.cif.gz, *e.g.* https://files-beta.wwpdb.org/wodnload/pdb_00001abc-sf.cif.gz
NMR data: restraints and chemical shifts	NEF, NMR-STAR and native refinement program formats	https://files-beta.wwpdb.org/pub/wwpdb/pdb/data/entries/[two-letter-hash]/[extended-PDB-ID]/experimental_data/	https://files-beta.wwpdb.org/download/[extended PDB ID].[NMR data format].gz, *e.g.* https://files-beta.wwpdb.org/download/pdb_00001abc_nmr-data.str.gz, https://files-beta.wwpdb.org/download/pdb_00001abc_nmr-data.nef.gz
Small-molecule references			
CCD	PDBx/mmCIF	https://files-beta.wwpdb.org/pub/wwpdb/refdata/chem_comp/[last-character-hash]/[CCD ID]/	https://files-beta.wwpdb.org/ligands/download/[CCD ID].cif, *e.g.*https://files-beta.wwpdb.org/ligands/download/ATP.cif
BIRD		https://files-beta.wwpdb.org/pub/wwpdb/refdata/bird, *e.g.* https://files-beta.wwpdb.org/pub/wwpdb/refdata/bird/prd/[last-character-hash]†	https://files-beta.wwpdb.org/birds/download/[BIRD ID].cif, *e.g.*https://files-beta.wwpdb.org/birds/download/PRD_000006.cif
Other derived data		https://files-beta.wwpdb.org/pub/wwpdb/refdata/derived_data/	
CCD holdings	JSON	A list of released chemical reference entries, their content types (*e.g.* Chemical Component, BIRD) and the most recent modification date of the reference file, https://files-beta.wwpdb.org/pub/wwpdb/refdata/derived_data/refdata_id_list.json.gz.	
SMILES, InChI, InChIKey	SDF, smi, inch		
Variants			
Assemblies	PDBx/mmCIF	https://files-beta.wwpdb.org/pub/wwpdb/pdb/data/entries/, https://files-beta.wwpdb.org/pub/wwpdb/pdb/data/entries/[two-letter-hash]/[extended-PDB-ID]/assemblies/	https://files-beta.wwpdb.org/download/[extended PDB ID]-assembly[number].cif.gz, *e.g.* https://files-beta.wwpdb.org/download/pdb_00001abc-assembly1.cif.gz
Archive holdings	JSON	https://files-beta.wwpdb.org/pub/wwpdb/pdb/holdings/	None, *e.g.*https://files-beta.wwpdb.org/pub/wwpdb/pdb/holdings/current_file_holdings.json.gz

† Note that paths are case-sensitive: use capital letters for hash and IDs.

**Table 2:** Resources and locations for extended PDB ID and PDBx/mmCIF

Resource types	Resources	Locations
FAQ	Extended PDB ID	https://www.wwpdb.org/documentation/pdb-id-extension-faq
FAQ	PDBx/mmCIF	https://mmcif.wwpdb.org/docs/faqs/pdbx-mmcif-faq-general.html
Dictionary	PDBx/mmCIF	https://mmcif.wwpdb.org
Tutorials	PDBx/mmCIF	https://pdb101.rcsb.org/learn/guide-to-understanding-pdb-data/beginner%E2%80%99s-guide-to-pdbx-mmcif
User guide	PDBx/mmCIF	https://mmcif.wwpdb.org/docs/user-guide/guide.html
Webinar	PDBx/mmCIF	https://pdb101.rcsb.org/train/training-events/mmcif
Webinar	PDB Beta Archive	https://pdb101.rcsb.org/train/training-events
Tools	*pdb_extract*	https://pdb-extract.wwpdb.org/
Tools	*CIF Editor*	https://pdbj.org/cif-editor
Tools	mmCIF parser and format conversion	https://mmcif.wwpdb.org/docs/software-resources.html
Tools	mmCIF *DB-Loader*	https://sw-tools.rcsb.org/apps/DB-LOADER/index.html
Tools	PostgresSQL database updater	https://gitlab.com/pdbjapan/mine2updater/
Download script and instruction	PDB Beta Archive	https://www.wwpdb.org/ftp/pdb-beta-ftp-sites
Archive	PDB Beta Archive	https://files-beta.wwpdb.org

## Data Availability

The PDB Beta Archive is accessible at https://files-beta.wwpdb.org. The PDBx/mmCIF dictionary is available at https://mmcif.wwpdb.org/.
